# How Perspectives on Food Safety of Vendors and Consumers Translate into Food-Choice Behaviors in 6 African and Asian Countries

**DOI:** 10.1016/j.cdnut.2022.100015

**Published:** 2022-12-22

**Authors:** Sejla Isanovic, Shilpa V. Constantinides, Edward A. Frongillo, Shiva Bhandari, Sharraf Samin, Emma Kenney, Sigrid Wertheim-Heck, Stella Nordhagen, Michelle Holdsworth, Paula Dominguez-Salas, Ramya Ambikapathi, Amos Laar, Crystal L. Patil, Bharati Kulkarni, Salome A. Bukachi, Mariah Ngutu, Christine E. Blake

**Affiliations:** 1 Department of Health Promotion, Education, and Behavior, Arnold School of Public Health, University of South Carolina, Columbia, SC, USA; 2 Environmental Policy Group, Department of Social Sciences, Wageningen University, Wageningen, the Netherlands; 3 Global Alliance for Improved Nutrition, Geneva, Switzerland; 4 Montpellier Interdisciplinary Center on Sustainable Agri-food Systems, University of Montpellier, French Agricultural Research Centre for International Development, International Center for Advanced Mediterranean Agronomic Studies, Mediterranean Agronomic Institute of Montpellier, French National Institute for Agricultural Research, Institut Agro, French National Research Institute for Sustainable Development, Montpellier, France; 5 Natural Resources Institute, University of Greenwich, London, United Kingdom; 6 International Livestock Research Institute, Nairobi, Kenya; 7 Department of Public Health, Purdue University, West Lafayette, IN, USA; 8 Department of Global Development, Cornell University, Ithaca, NY, USA; 9 University of Ghana, Department of Population, Family and Reproductive Health, School of Public Health, Accra, Ghana; 10 Department of Human Development Nursing Science, University of Illinois–Chicago, Chicago, IL, USA; 11 Division of Reproductive Biology, Maternal and Child Health and Nutrition, Indian Council of Medical Research, V. Ramalingaswami Bhawan, New Delhi, India; 12 Institute of Anthropology, Gender and African Studies, University of Nairobi, Nairobi, Kenya

**Keywords:** food policy, food safety, qualitative, consumer, Drivers of Food Choice, food choice, low- and middle-income countries, perspectives, supply chain, vendor

## Abstract

**Background:**

Consumption of unsafe foods increases morbidity and mortality and is currently an issue, particularly in low- and middle-income countries. Policy actions to ensure food safety are dominated by mitigation of biological and chemical hazards through supply-side risk management, lessening the degree to which consumer perspectives of food safety are considered.

**Objectives:**

This study aimed to provide an in-depth understanding, from vendor and consumer perspectives, of how food-safety concerns of consumers translate into their subsequent food-choice behaviors in 6 diverse low- and middle-income countries.

**Methods:**

Six Drivers of Food Choice projects (2016–2022) provided transcripts from 17 focus group discussions and 343 interviews conducted in Ghana, Guinea, India, Kenya, Tanzania, and Vietnam. Qualitative thematic analysis was used to identify emerging themes important to food safety.

**Results:**

The analysis suggests that consumers constructed meaning about food safety through personal lived experience and social influences. Community and family members contributed knowledge about food safety. Concerns about food safety were influenced by reputations of and relationships with food vendors. Consumers’ mistrust of food vendors was amplified by purposeful adulteration or unsafe selling practices and new methods used to produce food. Moreover, consumers were reassured of food safety by positive relationships with vendors; meals cooked at home; implementation of policies and following regulations; vendor adherence to environmental sanitation and food-hygiene practices; cleanliness of vendors’ appearance; and vendors’ or producers’ agency to use risk mitigation strategies in production, processing, and distribution of food.

**Conclusions:**

Consumers integrated their meanings, knowledge, and concerns about food safety to achieve assurance about the safety of their foods when making food-choice decisions. The success of food-safety policies hinges on consideration of consumers’ food-safety concerns in their design and implementation, alongside actions to reduce risk in food supply.

## Introduction

Rapid transitions in food systems in how food is produced, processed, and distributed are occurring in low- and middle-income countries (LMICs). Globalization, urbanization, income growth, climate change, and changes in consumer demand contribute to the changes seen in food systems [1] that may affect the availability and consumption of foods that are affordable, nutritious, and safe [2]. Within the context of LMICs undergoing urbanization, the expansion of food value chains (i.e., means of production and processing) provides individuals with more options, but the increased distance between food production and consumption may also result in more occasions for potential food contamination from poor handling, environmental conditions, inadequate sanitation, and cross-contamination [3]. Increasing levels of food contamination heighten the risk of contracting food-borne illnesses [1, 4]. In many LMICs, food-borne diseases are frequent and contribute to poor development outcomes and increased mortality rates, which can be further exacerbated when coupled with chronic poor dietary intake [5]. In 2019 LMICs represented 41% of the global population and accounted for 53% of all food-borne illnesses and 75% of related deaths [1]. Implementing food-safety regulations to mitigate the consumption of potentially hazardous foods is paramount.

Consumers attributed increases in disease prevalence to food-safety concerns, such as chemicals, contaminants, and adulteration, rather than concerns related to the nutritional content of the packaged food [6]. Barriers associated with purchasing safe food products included affordability [[6], [7], [8], [9], [10]], availability [7, 9, 11, 12], and the effectiveness of institutions regulating food safety [8, 13].

Literature on consumers’ contributions to economic change has predominantly represented consumers as passive recipients of the economy rather than as individuals who use economic goods and hold responsibilities, interests, and agencies [14]. Consumers, as active agents, have received little attention in the food economy, and the extent to which consumers’ perspectives of food safety may influence their food-choice decisions is unclear.

Consumers’ perspectives about food safety rely partly on the food’s smell, taste, attributes, and appearance as criteria for safe food consumption [9, 13, [15], [16], [17]]. Prinsen et al. [18] indicated that a food’s appearance held higher value than how it was processed and stored. Several studies focused on consumers’ perspectives of food safety found that specific subjective characteristics affect food-safety risk perceptions, including attitudes toward safe food purchasing and consumption [10, 19, 20], habits of safe food handling and consumption [9, 20], subjective norms for food-handling practices [[20], [21], [22]], self-efficacy of safe food purchasing and handling [17, 20, 21], expected positive outcomes of safe food preparation [21], knowledge and awareness of food-borne pathogens [11, 23], perceived control over safe food selection [17], and preferences for select food owing to food-safety concerns [10, 19]. Optimism bias, a tendency to underestimate one’s chances of experiencing a negative outcome, was associated with higher levels of education and decreased perceptions of risk [22, 24].

A recent systematic review of 46 studies in LMICs [7] reported that despite justifiable widespread food-safety concerns (chemical contamination, hygiene in and around food outlets, and unhygienic vendor practices), not all consumers could access or afford safe food. The review found that concerns about food safety could negatively influence consumers’ behaviors and diets and that consumers’ preferences for ultra-processed and packaged foods were influenced by concerns about food adulteration and vendor hygiene. Findings from the review demonstrated that concerns about food safety are linked to consumer dietary behaviors in LMICs, but how consumers’ concerns about food safety influence their food-choice behaviors and diet is not well understood.

This study aimed to provide an in-depth understanding, from vendor and consumer perspectives, of how consumers’ food-safety concerns translate into their subsequent food-choice behaviors. To address the aim, we posed 4 research questions: *1*) How do consumers construct meaning about food safety? *2*) Where do consumers obtain information and gain knowledge about food safety? *3*) How do consumers’ meanings and knowledge about food safety relate to their concerns about food safety? *4*) How do consumers integrate their meanings, knowledge, and concerns about food safety to engender assurance about the safety of their foods when making food-choice decisions?

## Methods

### Setting and sample

The Drivers of Food Choice (DFC) program funded 15 projects across West and South East Africa and South and South East Asia between 2016 and 2022. The projects generated evidence on the processes linking individuals’ decision making about food purchasing and consumption. The emergent data fomented the formation of the Food Safety Working Group, comprising the lead author (SI) and 6 coauthors (SC, EF, SB, SS, EK, CB). We identified the DFC projects that captured how food safety is conceptualized across different perspectives; some DFC projects did not have information on food safety because the projects that were funded in the DFC portfolio had different aims, not all of which covered food safety. Data from projects in the following 6 countries were included: Ghana [25], Guinea [26], India [27], Kenya [10], Tanzania [28], and Vietnam [29] (Table 1).

**TABLE 1:** Description of the 6 projects from the Drivers of Food Choice portfolio providing data for the study

Site	Urbanicity	Sample	Design	Method	How the study addressed food safety?
Ghana	Urban	Women; adolescent girls	Cross-sectional	Photovoice (*n* = 64)	Food hygiene, environmental sanitation, food adulteration, regulations
Guinea	Rural	Mothers; vendors	Cross-sectional	In-depth interviews (*n* = 89)	Food cleanliness, hygiene, food preparation
India	Peri-urban	Adult men and women; anganwadi worker (community health worker for maternal health and child development); farmers; village leaders; vendors and markets; shops and shopkeepers; banks	Cross-sectional	In-depth interviews (*n* = 57); focus group discussions (*n* = 10)	Concerns over quality and safety of fruit, perceived changes in the food environment, poor taste of food attributed to pesticides, skepticism around vendors
Kenya	Peri-urban	Adult men and women	Cross-sectional	In-depth interviews (*n* = 60); focus group discussions (*n* = 7); key informant interviews (*n* = 19)	Food sources and handling along the supply chain, contamination, concerns about vendors
Tanzania	Peri-urban	People living with HIV and their caregivers	Cross-sectional	In-depth interviews (*n* = 40)	Food-related strategies, constraints, issues affecting how caregivers feed people living with HIV, food environment
Vietnam	Urban	Main person responsible for food shopping	Cross-sectional	In-depth interviews (*n* = 14)	Food shopping practices and preferences, concerns, food environment mitigation strategies

Pradeilles et al. [25] used a community-based participatory method (Photovoice) to explore drivers influencing dietary behaviors. Participants were selected from neighborhoods in 2 cities in Ghana: Accra and Ho. Neighborhoods were chosen at random from a compiled list of low-income urban areas. Participants were purposefully recruited through communities, schools, and health services using quota sampling within the 2 selected neighborhoods: James Town in Accra (*n* = 62) and Ho Dome in Ho (*n* = 32). Male and female participants aged at least 13 y were selected based on demographic characteristics to ensure variations in participants’ views were captured (i.e., sex, age, body mass index, socioeconomic level, education level, and occupation status). Data were collected between May 2017 and June 2018 by members of the research team who were native speakers but not members of the selected neighborhoods. The Photovoice study was conducted on a subsample; a third of the participants were randomly selected from the larger sample. Participants were trained in using digital cameras and instructed to document their physical food environment (i.e., environments in which participants eat or acquire food). Ethical approval was obtained from the Ghana Health Service Ethics Review Committee (GHS-ERC 07/09/16 and GHS-ERC 02/05/17). The ethical committee granted permission for photographs to be reused in scientific outputs. Written informed consent was obtained from participants aged 18 y or older. Participants aged 13–17 y provided assent in addition to consent from their legal guardians. A photograph release form was used to request consent to take photographs if a person’s face was visible, and participants consented to photographs being used in scientific outputs. The data used in this study came from the Ghanaian subsample of participants selected for the Photovoice study [25].

In Guinea, Nordhagen et al. [26] used stratified sampling to purposively select study sites based on their proximal distance to villages and towns. Data were collected from May 2018 to December 2019 through nonparticipant observations at mining sites (*n* = 25) and markets (*n* = 8), household surveys (*n* = 613) and market surveys (*n* = 4), and in-depth interviews with mothers (*n* = 45) and food vendors (*n* = 40). Data collectors were familiar with the local context, fluent in the local languages, and trained rigorously in data collection methods. Households were randomly selected to participate in a household survey if a member was actively engaged in mining and had a child younger than 5 y living in the household. In-depth interviews were conducted with mothers of children younger than 5 y in mining households and food vendors from markets near the study sites. Interviews with mothers measured 4 dimensions of the food environment: accessibility, affordability, convenience, and desirability. Interviews with vendors measured 2 dimensions: convenience and desirability. This study was conducted according to the guidelines in the Declaration of Helsinki, and all procedures involving research study participants were approved by Guinea’s national research ethics council (Comité National d’Ethique et de Recherche en Santé, number 080/CNERS/18). Written informed consent was obtained from all subjects. The current study used data from in-depth interviews with mothers and vendors [26].

Surendran et al. [27] conducted 2 phases of data collection in Hyderabad, India. In phase I, the 2012–2014 Andhra Pradesh Children and Parents’ Study census was used to randomly recruit eligible household members from 2 villages, Patelguda and Thumaloor, to understand the general food environment. The 2 villages were selected based on their levels of urbanicity. For phase I, data collection occurred between June and August 2017 using semistructured in-depth interviews (*n* = 18). From each household, 1 man and 1 woman aged between 18 and 65 y were recruited using convenience sampling. Trained field workers from the Indian Institute of Public Health, Hyderabad, India, conducted the interviews. Phase II of the study collected data through focus group discussions to understand fruit and vegetable diversity choices across generations. Data collection occurred in August 2018. Eight villages were purposively selected based on levels of farm extensiveness. Convenience sampling selected participants based on availability during data collection and age range (i.e., 15–40 y, 41–65 y, and older than 65 y). Nine focus group discussions were conducted, comprising 8–16 participants. Trained field workers from the Indian Council of Medical Research, National Institute of Nutrition, India, led the focus group discussions. Field workers from both institutions were fluent in the local language, Telugu, and English. Field workers transcribed the recordings in Telugu and subsequently translated transcriptions into English. Ethical approval for phase I of the study was obtained from the ethics committee of the Indian Institute of Public Health, Hyderabad, under the Public Health Foundation India (reference number: IIPH/TRCIEC/092/2017) and the Observational Ethics Committee of the London School of Hygiene and Tropical Medicine (reference number: 12257). Ethical approval for phase II of the study was obtained from the ethics committee of the Indian Institute of Public Health, Hyderabad, under the Public Health Foundation India (reference number: IIPH/TRCIEC/170/2013-1-1-1-1-1) and the Indian Council of Medical Research, National Institute of Nutrition, India (reference number: CR04/0I/2017). The data used in the current study came from in-depth interviews and focus group discussions [27].

In Kenya, Bukachi et al. [10] conducted a study in low-income informal settlements in 6 wards in the North and South Dagoretti subcounties. Data were collected from December 2019 to February 2020 through in-depth interviews (*n* = 60), focus group discussions (*n* = 19), and key informant interviews (*n* = 19) by trained data collectors. Participants were purposively sampled based on the study’s inclusion criteria (i.e., men and women of reproductive age who lived together in a couple-based family with a child aged <5 y). Participants were identified through local community health workers. Key informant interviews were conducted with purposively sampled informants in the study area. Participants were recruited for key informant interviews based on the extent of their health and nutrition knowledge. Questions about food acquisition, choices, preparation (i.e., methods, time, and processes), consumption patterns, and decision making guided in-depth interviews, focus group discussions, and key informant interviews. The study received ethical approval from the International Livestock Research Institute Institutional Research Ethics committee (number ILRI-IREC 2018/16/1). Written informed consent was obtained from all participants. The current study used the same data set [10].

In Tanzania, Boncyk et al. [28] described an analysis of face-to-face semistructured interviews (60–90 minutes) with people living with HIV (PLHIV; *n* = 20) and family members caring for PLHIV (*n* = 20). Interviews were conducted from July 2019 to March 2020 in Kiswahili by an experienced Tanzanian qualitative researcher. The PLHIV interview guide included questions about food choices and consumption and procurement and processing since HIV diagnosis. Interviews with family members caring for PLHIV (*n* = 20) mirrored the PLHIV guide with specific questions about accommodating to food preferences of PLHIV. This study was approved by Institutional Review Boards at Purdue University (USA, #1806020735) and the National Institute of Medical Research (Tanzania, #2899). All participants provided informed consent. To ensure confidentiality and anonymity, all potential identifiers were replaced with pseudonyms. The current study used the same data set [28].

Two low-income urban districts in Hanoi were selected as the study areas in Vietnam [[29], [30], [31]]. Geographic areas within the 2 districts were stratified based on the availability and accessibility of supermarkets and formal wet markets within walking distance of participants’ households. Quantitative and qualitative data collection occurred from November 2017 to October 2018 [[30], [31]]. Experienced data collectors with rigorous training in data collection methods conducted the qualitative interviews. Households were randomly sampled in each stratum using a door-to-door sampling strategy. The qualitative study was conducted on a subsample of households; of the 35 households selected, participants from 14 households were included. In-depth interviews were conducted with the primary respondents identified from the larger sample (women of childbearing age or born after 1966, primarily responsible for household food acquisition) and their mothers (-in-laws) living in the same household. The interviews explored patterns of everyday food consumption practices over time and across generations [30]. Researchers obtained full informed verbal consent from all participants. The study received ethical approval from the Hanoi Medical University, Vietnam, in June 2017 (IRB00003121). The data used in the current study came from in-depth qualitative interviews [29].

The 6 project study sites differed by urbanicity, that is, 2 urban [Ghana [25] and Vietnam [29]], 3 peri-urban [India [27], Kenya [6], and Tanzania [28]], and 1 rural [Guinea [26]] (Table 1). For the data used in this study, samples across the 6 projects comprised women and adolescent girls [Ghana [25]], caregivers and mothers of children younger than 5 y, and food vendors [Guinea [26]], men and women with children younger than 5 y [Kenya [6] and India [27]], individuals living with HIV and their caregivers [Tanzania [28]], and individuals responsible for household food purchases [Vietnam [29]] (Table 1). These 6 studies used cross-sectional study designs.

Principal investigators from each project conducted a preliminary review of their data to extract transcripts that addressed food safety (Table 1). Projects in Ghana, India, Kenya, and Tanzania provided complete transcripts translated to English. Principal investigators provided excerpts from transcripts for the Guinea and Vietnam projects on select questions and responses related to food safety. For the Vietnamese project, the local research team members completed the Vietnamese to English translation of the transcripts. The DFC Food Safety Working Group hired a translator fluent in Guinean French to translate the Guinea transcript segments.

### Data analysis

Preliminary coding of the data was conducted to build the codebook. The codebook underwent revisions until all emergent codes were identified. Subsequent modifications helped clarify code descriptions and classifications to finalize the codebook.

The 6 projects provided transcripts from 17 focus group discussions and 343 in-depth interviews (including 64 interviews that used Photovoice). Of these 360 transcripts, 305 contained data on food safety (17 focus group discussions and 288 interviews). Transcripts excluded from the analysis (72 interviews) included duplicates (3) and transcripts that did not contain information related to food safety (69). Projects from 4 countries (Ghana, Kenya, Tanzania, and India) provided complete transcripts, whereas projects from 2 (Guinea and Vietnam) provided translated transcript segments.

A qualitative thematic analysis was used to identify themes important to food safety. We used the 6-phase framework of Braun and Clarke [32] to guide the process of the thematic analysis: *1*) becoming familiar with the data, *2*) generating initial codes, *3*) searching for themes, *4*) reviewing themes, *5*) defining themes, and *6*) reporting themes. Two analysts (SI, SC) trained the Food Safety Working Group members (SB, SS, EK) on identifying codes using the codebook.

Five members (SI, SC, SB, SS, EK) of the Food Safety Working Group conducted the first cycle of coding. During the first coding cycle, codes were determined on a semantic level, capturing the surface meaning of the data [33]. This coding decision was made considering the researchers’ positionality and challenges in interpreting the data resulting from translations and cultural differences [34]. Complete transcripts were coded from 4 countries (Ghana, Kenya, Tanzania, and India), and the translated transcript segments were coded from 2 (Guinea and Vietnam). A random number generator was used to select 20% of the full transcripts to be coded by 2 analysts. Two analysts coded all translated segments from Guinea and Vietnam. The coding team met weekly to review coded transcripts and identify and resolve discrepancies between coders. The lead analyst reviewed all coded transcripts to ensure data were coded accurately.

The lead analyst (SI) conducted the second coding cycle, categorizing the first cycle of codes into themes. Developing themes consisted of sorting the codes and collating relevant coded data extracts to the identified themes. The finalized themes demonstrated meaningful coherence between data, representing internal homogeneity, and clear, identifiable distinctions between themes, representing external heterogeneity [32]. Codes listed under each theme were categorized to form subthemes. Pattern coding identified subthemes from the transcript data [35]. The subthemes organized similarly coded data and described the conditions and characteristics of each theme.

Transcripts were coded in Microsoft Word using the comments feature. Then, the extracted codes and corresponding text segments were converted from a Word document to an Excel document using a program written in Python 3.10.1. Matrices were developed for each project to tabulate the following for extracted codes: *1*) double coded (yes/no); *2*) coder and double coder, if applicable; *3*) transcript document label; *4*) code; *5*) text segment; and *6*) interviewee demographic information including age, sex, occupation, socioeconomic status, and education. Available demographic information varied with each project.

## Results

Fourteen themes related to food safety emerged from the data: *1*) constructed narratives from personal lived experience, *2*) constructed narratives from social influences, *3*) sources of information, *4*) vendor relationship and reputation, *5*) vendor appearance, *6*) purposeful adulteration and unsafe selling practices, *7*) environmental sanitation, *8*) food-hygiene practices, *9*) transparency of meals cooked at home, *10*) vendors’ or producers’ agency, *11*) trust or mistrust in implementation of policies and regulations, *12*) mistrust of new methods used to grow and process foods, *13*) transparency of food processes in the food supply chain, and *14*) inadvertent contamination of food (Table 2).

**TABLE 2:** The 14 emergent themes about perspectives of food safety and their descriptions

Theme	Description
Constructed narratives from personal experience	A way for consumers to construct their interpretation of food safety. These narratives are rooted in one’s ideologies, everyday practices, personal experiences, and ways of thinking and provide consumers with insight or an understanding of what food safety means to them, including related practices, processes, and consequences.
Constructed narratives from social influences	A way for consumers to construct their interpretation of food safety. These narratives are influenced by their culture, religion, rituals, and social traditions. These narratives help the consumers shape their understanding of food safety, including related practices, processes, and consequences.
Sources of information	Consumers reported receiving information about food safety (whether valid or false information) from sources such as media (e.g., television, radio), health care workers (e.g., health clinic staff, doctors, nurses), teachers, and peers (e.g., family members, friends).
Mistrust of new methods used to grow/process food	Consumers’ mistrust of new methods and technologies used during food production and harvesting (i.e., use of pesticides, bioengineered genes, fertilizers, antibiotics, growth hormones, and by-products).
Inadvertent contamination of food	Consumers cited concerns about foods coming into contact with chemicals or other contaminants from pesticide or sewage runoff, owing to the location of where food is produced in relation to the application or contaminated site.
Vendor relationship and reputation	Consumer’s belief that vendor’s food is safe or unsafe is contingent on the relationship that forms from previous experience or vendor’s reputation. The vendor’s reputation is verified by the community and (in)validates the source and quality of food.
Purposeful adulteration or unsafe selling practices	Consumers’ mistrust of vendors stemmed from concerns of changes in taste and appearance of food due to added substances meant to prolong shelf life and exposure of food malpractice (i.e., relabeling expired foods and reselling foods). Consumers indicate health consequences associated with the unsafe selling practices.
Transparency of process in food supply chain	Consumers were influenced to believe that food was safe or unsafe depending on their trust or mistrust in each of the stages and the types of roles involved (e.g., farmers, distributors, retailers) during the production, processing, and distribution stages within the food supply chain.
Vendor’s appearance	Consumers were influenced to believe food was safe or unsafe depending on observable cleanliness of vendors (e.g., dressed in appropriate clothing: hairnets, gloves, aprons without visible stains, and sweat).
Environmental sanitation	Consumers were influenced to believe food was safe or unsafe depending on the physical environment around the shop or retail area and food area (e.g., presence of litter and flies).
Food-hygiene practices	Consumers were influenced to believe food was safe or unsafe depending on practices followed when preparing food (e.g., handwashing practices, use of clean or unclean water, foods covered to ensure cleanliness, washing foods, and cleanliness of dishes).
Transparency of meals cooked at home	Consumers felt food prepared at home to be safer than meals obtained outside of the home, based on the hygiene practices applied (i.e., handwashing practices, covering foods, and washing fruits or vegetables).
Vendors’ or producers’ agency	Consumers and vendors believed that the quality and safety of their food was validated by their utilization of risk mitigation strategies, such as their capacity to trace and control the products, ingredients, supplies, and processing operations included throughout the food production chain. (i.e., one trusts their ability to acquire, process, and prepare food safely; their family eats the same foods they sell, they use the same technique to prepare foods for consumers as they would for themselves, they have control over the foods sold, and the location of their shop).
Trust or mistrust in implementation of policies and following regulations	Consumers' confidence in the safety of their food depended on the local food system's ability to enforce and enhance quality control, consumers' ability to inspect food to determine its safety (e.g., checking the expiration date or package quality), and vendors' ability to follow food safety rules.

Themes permeated highly across the 6 projects, despite the differences in the samples and methods. For example, vendors and consumers from every site reflected on vendor relationships and reputation, purposeful adulteration or unsafe selling practices, food-hygiene practices, and trust or mistrust in the implementation of policies and following regulations. Other themes were shared among most sites, such as constructed narratives from personal lived experiences (Ghana, India, Kenya, Tanzania, and Vietnam), sources of information (Ghana, India, Kenya, Tanzania, and Vietnam), environmental sanitation (Ghana, Guinea, India, Kenya, and Tanzania), transparency of meals cooked at home (Ghana, Guinea, India, Kenya, and Tanzania), and mistrust of methods used to grow and process foods (Ghana, India, Kenya, Tanzania, and Vietnam) (Table 3).

**TABLE 3:** Appearance of themes expressed as a percentage of total number of transcripts in each country

Theme	Ghana (*n* = 64)	Guinea (*n* = 63)	India (*n* = 44)	Kenya (*n* = 82)	Tanzania (*n* = 39)	Vietnam (*n* = 14)
Constructed narratives from personal experience	17	0	25	62	46	57
Constructed narratives from social influences	2	0	34	16	26	0
Sources of information	34	0	16	40	38	14
Mistrust of new methods used to grow and process foods	5	0	61	38	46	64
Inadvertent contamination of food	2	0	0	12	36	0
Vendor relationship and reputation	48	41	2	65	28	79
Purposeful adulteration or unsafe selling practices	14	2	7	70	21	29
Transparency of process in food supply chain	0	11	0	32	10	14
Vendor’s appearance	14	65	0	33	13	0
Environmental sanitation	75	73	2	56	33	0
Food-hygiene practices	69	87	45	78	59	50
Transparency of meals cooked at home	42	2	2	18	15	0
Vendors’ or producers’ agency	0	13	7	9	8	0
Trust or mistrust in implementation of policies and following of regulations	9	2	5	37	5	43

### How do consumers construct meaning about food safety?

Consumers in Ghana, India, Kenya, Tanzania, and Vietnam constructed meanings about food safety through narratives based on personal experiences and social influences. Foods deemed unsafe for consumption were attributed to external forces compromising food safety. For example, a respondent in Vietnam reported direct experiences witnessing unsafe food-handling practices at 2 supermarkets that caused her to doubt the processes used to test and certify food safety and question which retailers she could trust (Table 4). Experiences witnessing unsafe practices in food production and retail, such as farmers using chemicals in agriculture and vendors selling expired foods, were seen as compromising food safety.

**TABLE 4:** Reports of vendors’ and consumers’ perspectives on food safety, categorized by theme

Theme	Example quotations
How do consumers construct meaning about food safety?	
Constructed narratives from personal experience	R: “Although your vegetables is dirty, but it still can be recognized as 100% clean if you used your money to lobby. The society now is like that. It is not transparent. So, it’s hard for me to say the food is safe or not, even if foods was tested. I only trust if it is foods from my family. I was dissatisfied with the supermarket when I saw that. I feel so upset about these 2 supermarkets. But lay people do not have the voice to complain and give feedback.” (Vietnam)
	R: “They are growing gardens using sprays and all so for that medicine effect to be washed away, we are putting in the salt water.” (India)
Constructed narratives from social influences	Culture
	I: “Are there any superstitions in the matter of taking food?” R: “Yes… Pregnant women do not eat papaya and banana fruit because when they eat papaya, it will cause abortion and if they eat banana then newborn baby will have breathing problems.” (India)
	I: “With you being pregnant right now, does your Auntie advise you to eat certain foods and avoid others?” R: “What she normally tells me is that I should not be sitting outside to eat because it is not everyone who thinks well of you. You know someone could even look into my food with an ‘evil’ eye so the food I am eating harms the baby I am carrying.” (Ghana)
	Religion
	*Pork* R1: “The bible prohibits it […] The second thing, pigs eat all the dirty things that they come across. […] R2: “We do not eat pork, it has been refused […] In the bible it is written or has been refused.” R1: “Demons were chased into them.” (Kenya)
	*Offal* R: “Akorinos believe that all the organs […] involved in a circulatory system that is all the organs where blood passes, […] those organs plus blood should be disposed of and if they are not, they cannot take or eat those parts. Also they believe if the animal is taken with all those organs, together with the blood also they cannot eat that, yeah. They cannot eat because of their religious beliefs.” (Kenya)
Where do consumers obtain information and gain knowledge about food safety?	
Sources of information	Health care system
	*Hospital* R: “When you go to the hospital you are asked what did you eat yesterday, you tell them I ate meat; they tell you that meat had a problem.” (Kenya)
	*Nurse* R: “So I listen to the education given at the hospital and I eat based on that.” (Ghana)
	Media
	*TV* I: “So, who told you madam that if you use medicine on the crop, it is not good and it is harmful?” R: “On TV, in news they will tell…” (India)
	*Radio* R: “How do you know its origin when they only wrap vegetable by nylon pack. Even now there is origin traceability in the supermarket—stamps, or labels. But to say that I really feel safe, the answer is not yet. Because there are also supermarkets having bad news on the radio.” I: “What is the case?” R: “In the past, newspaper, radio, and TV reveal that there were too many cases in the supermarket that expired goods were relabeled and sold as normal.” (Vietnam)
	Social networks
	*Community* R: “Nowadays people are not very sure if the meat they are eating is animal or it belongs to a human being, we hear at times that human meat has been found in a butchery.” (Kenya)
	*Family* R: “Our grandfather told us not to eat kenkey. The reason our grandfather gave was that the way kenkey is prepared is usually not in a hygienic condition.” (Ghana)
	*Family* R: “He will advise me that and tell me that it is not good. If I am going to cook such things, he tells me not to cook them. Or he will show me the way I can use them, then he will teach me before I will cook it and eat.” (Ghana)
How do consumers’ meanings and knowledge about food safety relate to their concerns about food safety?	
Mistrust of new methods used to grow and process food	R: “At that time there weren’t these many pesticides, only crops were grown with manure, same crops, but they used to use manure, there weren’t fertilizers. Now manure they are using and fertilizers also they are using equally. Now diabetes, BP, thyroid, cancer all diseases coming, why it is coming you should know. All that we are cultivating, they are going into our stomach, somebody who ate is getting diseases.” (India)
Inadvertent contamination of food	R: “It is dirty water in general from latrines or dirty sewages, and about spinach I worry because they sprout so fast to the point I wonder, I wonder how is that.” (Tanzania)
Vendor relationship and reputation	R: “You know there are other waakye sellers around and they don’t prepare the food in hygienic conditions. They are also sold close to the gutter and there are stones in the food so I prefer to buy at this particular food vendor.” (Ghana)
Purposeful adulteration or unsafe selling practices	R: “One time there was a woman who was telling us that the milk has not expired but if you look closely you find that there are 2 expiry stickers on the package so even when it has expired, they remove the first sticker so that it seems as if it has not yet expired.” (Kenya)
Transparency of process in food supply chain	I: “Even though you buy pork from the familiar vendor, you still need to check it?” R: “Yes. He does also buy from the producers, he does not feed the pigs by himself. Hence, he may not know about the safety of the pigs. If the pigs are not safe but producers tell the lie, he will still believe in it. However, generally speaking, in Vietnam, producers and sellers do not care for consumers, they just care for the profit.” (Vietnam)
	R: “Safety issues can arise at any level. Like at the production level you may find that a person is taking a sickly cow to the slaughterhouse, and then at the slaughterhouse if the sickly cow is not inspected it will be sold to the retailers and that will be bad. At the retailer level like me you may find that maybe the retailer is selling meat that has overstayed, and also some unhygienic practices. And at the consumer level you may find also unhygienic practices and also the person has not cooked well […] In short, everyone has a part to play when it comes to safety.” (Kenya)
Mistrust in implementation of policies and following of regulations	R: “Instead of using Maggi cubes, they will use A1, and this A1 thing is more dangerous to our health. I know it has been banned, but it is still on the market.” (Ghana)
	R: “I wish the Accra Metropolitan Assembly authorities will come and inspect where people prepare food before giving them the license to cook. In Jamestown, anyone wakes up and start to sell kenkey.” (Ghana)
	R: “I think it needs to be something that confirms the safety level of food so that I can trust and feel secure of what I buy for the family. There should be a measure to prove that it is really safe, […] then when I buy it and know it is fresh, but I still have the feeling of worry.” (Vietnam)
	R: “When you go to town, where this meat has been prepared well, inspected, and found it to be fit for consumption, but here we can’t know where it was inspected. Whether it was inspected or not, […] when you go there, you will get them with stamps, but in slums, you don’t.” (Kenya)
	R: “For safety, I would like to know if the meat is well inspected because maybe you might see the stamp due to corruption and that meat might not be fit for human consumption.” (Kenya)
How do consumers integrate their meanings, knowledge, and concerns about food safety to engender assurance about the safety of their foods when making food-choice decisions?	
Vendor’s appearance	R: “There is a seller that I trust because she is clean, I do not buy because I like her, but because of her.” (Guinea)
Environmental sanitation	R: “I like the way she keeps the surroundings so neat. So, once you eat at a neat place, you will not fall sick. But if there is a gutter around and it is not covered and flies from the gutter come and land on your food, you can get Cholera. So, to avoid all this, I like buying food from her because her place is always neat.” (Ghana)
Food-hygiene practices	R: “The signs that show that the food is healthy—when the saleswoman is clean by her clothes, the plates are clean, she washes them with soap.” (Guinea)
	R: “There is a specific butchery where I go to purchase meat. I like the butchery because of its outlook. There are several butchers at our place, other butchers use machete to cut meat but this one cuts meat with a machine. Using a machine is good because it doesn’t involve touching the meat frequently.” (Tanzania)
Transparency of home-cooked meals	R: “Someone cooking outside I do not know the kind of hygiene she has… Maybe she has not washed her hands, but she has been cutting onions with her dirty hands. I will be affected at the end of the day, but when I am cooking at home, I will wash my hands, I will wash the vegetables, I will get the hygiene.” (Ghana)
Vendor relationship and reputation	R: “It may be that if I know a place where it is safe and well-nourished, then I’m willing to come and buy from there and not always convenience in first place.” (Vietnam)
Vendors’ or producers’ agency	R: “When I prepare the meal I sell, I take part of that meal for my family’s food. So my family eats what I sell. With this, there is no doubt about the quality of hygiene of the meal that I sell.” (Guinea)
Trust in implementation of policies and following of regulations	R: “Rules are strict due to government oversight, or the veterinaries from the government makes sure whatever products comes from there is very safe. Also, you see we need business permits…” (Kenya)
	R2: “This is the Van Noi clean vegetables cooperative in Dong Anh district. They must have a certificate, if you want to check it, just pass by there.” R1: “That is the vegetables, which you can verify the origin by visiting their farm.” (Vietnam)

Consumers associated health consequences with consuming unsafe foods and reported the symptoms they experienced, including ill stomach (e.g., diarrhea and vomiting) and chronic pain, from consuming unsafe foods. Descriptions of cultural taboos seemed to be embedded within the context of food safety. Consumers conveyed similar health-related concerns about consuming foods deemed unsafe by their cultural beliefs (i.e., food taboos). Respondents in Ghana, India, and Kenya indicated that children and pregnant mothers were at higher risks of contracting food-related illnesses if they consumed foods that were considered taboo within their context. For example, a respondent from India described food restrictions directed toward women during pregnancy to avoid adverse events such as miscarriage and respiratory issues (Table 4). Some respondents spoke of how social taboos limited where women could consume foods during pregnancy, such as a woman from Ghana who was instructed to avoid consuming foods in public settings for fear of pregnancy complications caused by onlookers projecting negative thoughts onto her food (Table 4). Similarly, perspectives of food safety were nested in religious beliefs. Religion was a form of instruction that helped interpret which foods were fit or unfit for consumption. Religious prescriptions were informed by unsanitary conditions, as mentioned by study participants (Table 4).

The practices that consumers learned to apply to prevent consuming contaminated foods varied based on their daily experiences witnessing unsafe food-handling practices and the consequences of consuming unsafe foods. Some consumers described strategies they learned to minimize agrochemical exposure, such as soaking and washing foods and strictly eating home-cooked meals (Table 4). Others reported avoiding specific locations and vendors whose foods they deemed unsafe for consumption based on previous encounters.

### Where do consumers obtain information and gain knowledge about food safety?

Consumers from Ghana, India, Kenya, Tanzania, and Vietnam reported health care systems, media, and social networks as important sources of information about food safety. Respondents from Ghana, Guinea, India, and Kenya who sought care for food-related illnesses at hospitals and clinics reported learning about the food source contributing to their sickness (Table 4). Few respondents received instructions on food-safety practices, such as maintaining hygienic conditions and ensuring food is cooked thoroughly; however, most respondents were told to avoid consuming the food that caused their illness.

Across contexts, respondents cited television and radio sources as media sources that contributed to their knowledge of food safety. The 2 sources relayed information on adulterated foods, exposing retail establishments selling low-quality products, processing units following poor hygiene and environmental sanitation practices, and cultivators using agrochemicals to produce foods (Table 4).

Much of the information shared within community-based social networks was linked to adulteration. Consumers often viewed animal-source foods, such as milk and meat, as the most frequently adulterated foods. Respondents described stories circulating in their communities about butcheries deliberately selling diseased meat and grocery stores altering meat products to appear fresh (e.g., using preservatives). Consumers in Kenya described limiting and, at times, avoiding consuming meat products, such as chicken and beef, owing to the fear incited by local accounts (Table 4).

Knowledge about food safety was shared among families. Family members communicated mistrust in vendors’ hygiene practices and guidance on where to purchase foods deemed safe for consumption. Consumers were informed of vendors’ poor hygiene practices by older family members, frequently citing their mothers (in-laws) and grandparents as sources. Furthermore, family members shared information on strategies for determining which vendors to purchase foods from, referencing environmental sanitation cues that pose potential contamination risks, such as surrounding flies and littered retail spaces. In addition, family members offered guidance on where to buy safe food and what safe food-handling practices to follow when preparing foods (e.g., cleaning hands, surfaces, and utensils) (Table 4).

### How do consumers’ meanings and knowledge about food safety relate to their concerns about food safety?

Across all contexts, consumers cited concerns of food producers and retailers following food practices that threatened the safe distribution of foods. According to consumers, practices that prompted concerns included untrustworthy methods used to grow and process foods, inadvertent contamination of foods, purposeful adulteration, unsafe selling practices, and lack of transparency throughout the food production and processing phases. Consumers were concerned about whether vendors followed safe food practices based on observations of the retail location, the environment around the retail space, and the vendor’s hygiene practices. Consumers familiar with the health consequences of consuming contaminated foods shared their concerns about food safety, such as contracting diseases from polluted environments.

Consumers from Ghana, India, Kenya, Tanzania, and Vietnam were skeptical about the methods used to grow and process foods owing to the widespread use of chemicals in crops and animal-source foods. Consumers reported being wary of how the farmers’ use of agrochemicals, such as fertilizers and pesticides, to grow crops would affect their health. Consumption of livestock injected with antibiotics and growth hormones induced similar concerns among consumers (Table 4). Foods contaminated with chemicals such as antibiotics, fertilizers, growth hormones, and preservatives were often connected to poor health outcomes, such as chronic illnesses (e.g., cancer), a shorter life expectancy, and decreased energy and strength.

Inadvertent food contamination throughout the supply chain was another concern about food safety. These concerns focused on the nearby environment where food was grown and sold. Consumers questioned the safety of foods grown near sewage; they frequently spoke of health consequences from contact with foods exposed to toxins (Table 4). Concerns about food contamination in retail related to vendors’ proximity to contaminated sites, such as gutters or sewage plants.

Consumers attributed their fears of food malpractice to food retailers using food additives to increase foods’ appeal, uncleaned containers to pack and store foods, and false labels to relabel expired foods (Table 4). Consumers who witnessed vendors working in unsanitary environments or neglecting to follow food-hygiene practices believed those vendor-sold foods were unsafe for consumption. Consumers felt vendors’ poor hygiene practices were reflections of their intentions and assumed those vendors were willing to compromise food safety for profit.

Consumers expressed concerns about the transparency of the food supply chain through the production, processing, and retail phases. Consumers reported being skeptical about the safety of the food source (Table 4) and questioned whether retailers followed hygienic practices while storing, distributing, and selling foods. As consumers identified the points at which food safety was likely to be compromised, they also recognized the breadth and depth of health consequences that could ensue, indicating that risks to food safety can occur at all levels across the production, distribution, selling, purchasing, and consumption phases. Moreover, consumers identified the stages at which unsafe food practices can occur and what that may mean for exposure rates; unsafe food practices occurring at the production and processing phases could affect an entire community compared with unsafe practices at the household level (Table 4).

Concerns about food safety created mistrust in the implemented policies addressing food safety. Consumers cited the authorities’ lack of commitment to fighting corruption, describing instances of witnessing banned products on the market or approval of vendor licenses without conducting regulatory inspections. Consumers described policies and regulations on food safety as unreliable, reporting inconsistencies in food inspections and classification of safety standards (Table 4). In Ghana and Kenya, consumers demanded government involvement to enable the development of higher standards in hygiene practices and fairness in regulation.

### How do consumers integrate their meanings, knowledge, and concerns about food safety to engender assurance about the safety of their foods when making food-choice decisions?

Consumers believed food was safe for consumption after assessing the vendor’s environment and food-hygiene practices. Vendors who prepared hygienic foods in a sanitary environment were trusted to provide safe food. Consumers felt comfortable purchasing foods from vendors provided they wore appropriate workwear, clean aprons, and gloves while handling food, especially meat and meat products (Table 4). A “clean” appearance implied that the vendor worked in a safe environment and followed hygiene practices. Relational aspects, such as positive interactions between the vendor and consumer, where the vendor was perceived as welcoming, assured consumers they could trust the safety of the food. In addition, consumers pointed to cues in retail that reassured their food was safe for consumption, such as food inspectors’ presence around retail outlets and evidence of food inspection through certification stamps and package labeling.

Findings from vendor interviews conducted in Guinea were congruous with the results reported by consumers about methods used to ensure food was safe for consumption. Vendors reported practicing personal hygiene (e.g., handwashing and wearing clean clothes), cross-contamination prevention, proper storage and cooking, and environmental sanitation (Table 4). Responses among vendors concentrated on hygiene practices, such as maintaining cleanliness, avoiding contamination, and maintaining control over the production and preparation of food. Vendors acknowledged following hygiene guidelines, regulatory food procedures, and inspections to ensure safety.

Respondents felt food cooked at home was safer than prepared food purchased from vendors on account of hygiene and environmental sanitation practices applied (Table 4). Respondents projected confidence in their cooking practices, attributing their knowledge of safe food preparation to a lower risk of contracting food-related sicknesses, unlike purchasing prepared foods from vendors. Respondents felt cross-contamination could be prevented by cleaning the designated cooking area and using clean dishware.

Coupled with visual cues consumers use to indicate a vendor’s cleanliness, consumers from Ghana, Guinea, Kenya, and Vietnam also perceived positive interactions with vendors as an indicator of the vendor’s character. Some consumers indicated that a vendor’s welcoming demeanor reinforced their trust in the safety of the vendor’s food, provided the vendor followed food-hygiene and environmental sanitation practices (Table 4). Other consumers felt they could trust the vendors recommended to them by family members.

Connections were identified linking consumers’ trust in the safety of purchased foods to the implemented food-related policies and following regulations. Consumers considered decreased reports of food-borne illnesses and increased presence of inspection officers as evidence of food retailers following food-safety guidelines. Cues from vendors helped consumers confirm that food-safety regulations were observed (e.g., vendors following environmental sanitation standards and signage, such as stamps and food labels offering evidence of food inspection) (Table 4).

## Discussion

From the qualitative thematic analysis to capture vendors’ and consumers’ food-safety perspectives across 6 diverse study sites in LMICs, 14 themes emerged, reflecting how consumers’ experiences within the context of their environments construct and shape their understanding of food safety, ultimately influencing their food choices. Our findings provide insight into how consumers construct meanings about food safety, where they receive information that shapes their understanding of food safety, how their understanding relates to their concerns, and how consumers integrate their meanings, knowledge, and concerns about food safety to achieve reassurance regarding the safety of their food.

Sensory level changes in foods familiar to consumers were often attributed to food malpractice. Differences in the food’s sensory features influenced consumers to believe the food’s perceptual features were compromised, particularly regarding the nutritional content, health value, and quality. Leng et al. [36] and Wertheim-Heck et al. [17] identified similar findings: consumers’ first impression of the food’s properties was reason enough to sow ideas of trust or mistrust in food vendors’ abilities to uphold food safety.

Cognitive processes shape skills, knowledge, attitude, liking and preference, anticipated consequences, and personal identity [37]. Perceived knowledge is critical in explaining food-choice variation [38]. A consumer’s decision-making process concerning food choice uses evaluation-based components such as attitude, liking, and preference [3, 17, 39]. Some consumers assessed the safety of food items through sensory evaluations and described their aversion to purchasing foods near contaminated sites [17]. Consumers reported a preference for purchasing foods from specific shops, citing previous vendors’ proximity to contaminated areas, indicating consumers conduct food-safety evaluations through comparison [16, 37, 40].

Consumers’ habits and experiences influence the narratives they construct about food safety [16]. Studies have suggested the importance of understanding the role of habitual patterns in shaping food-choice preferences [3, 20, 36, 41]. Consumers’ routines and habits around food consumption practices contributed to their understanding of food safety [3]. Understanding how consumers come to interpret food safety through their daily interactions and activities demonstrates the importance of considering the role of experiences and habits in decision-making processes [3].

In addition to the information consumers retrieve from food items, such as the brand, label, and packaging [40], consumers rely on their social environment (e.g., social networks and media outlets) to inform them which foods are safe to consume. These findings indicate that the social environment plays an influential role in shifting consumers’ perspectives about food safety, whether through raising concerns about phases of food production and processing or disseminating knowledge about mitigating food-safety risks [16, 37].

The physical environment (e.g., retail conditions, vendor appearance, food-hygiene practices, and environmental sanitation) provided consumers with information regarding the vendor’s adherence to food-safety regulations. Mistrust in food vendors’ ability to follow safe food practices led consumers to buy prepackaged foods, equating fewer instances of food contamination to healthiness. Nordhagen et al. [9] and Pradeilles et al. [25] found that consumers not only perceived the risk of exposure to contaminants such as bacteria as being lower with prepackaged foods but also noted that many of these foods are poor in nutrients. They contain high fat and sugar contents, which are associated with the risk of noncommunicable diseases [42, 43]. This study reinforces the findings from Nordhagen et al. [9] regarding the tendency of consumers to use binary thinking about the safety of foods and, in this particular context, associate processed or packaged foods as safer for consumption than unprocessed foods [44]. The results highlight the influence of the broader context (i.e., the retail food environment) on consumers’ food choices. Within this context, the process through which consumers experience their environment shapes the meanings they associate with foods in their environment, subsequently influencing their food-choice behaviors [45].

Consumers viewed food policies and regulations as unresponsive to their concerns. The views of consumers in this study are in accordance with those found in other studies indicating that poor implementation of regulations and measures promoting food safety can increase consumers’ concerns and inhibit feelings of reassurance about the safety of their foods [46, 47]. Not considering consumers’ experiences and perspectives, which are influenced by their values and beliefs, is one potential reason why policies may not have provided consumers with reassurance about the safety of their food [48, 49].

This analysis of vendors’ and consumers’ perspectives on food safety supports recent advances in the literature documenting the importance of understanding individual food choices for developing and improving food-related interventions [50]. Food-choice processes derive from the consumer’s experiences and are specific to their context [51, 52]. An individual’s dynamic nature is reflected in their decision-making processes, with shifts occurring throughout their life course [53, 54]. This study highlights the complex nature of individual decision making in the context of food safety. Given what is known about the multiple levels of influence of food choice [45], we expect consumers’ perspectives on food safety to form through similar interactions across the personal, social, and environmental levels of influence.

This analysis used data from projects implemented in parts of Africa and Asia that provided information about food safety. Although the samples used in this study were not representative of all LMIC populations, the projects that contributed data were diverse regarding the sample demographics, urbanicity, and geographic location (Table 1). Although the projects from which data were used for this analysis did not explicitly seek to assess consumers’ food-safety perspectives, evidence of the prominence of topics related to food-safety perspectives reinforces the importance of engaging in consumers’ perspectives of food safety for policies and interventions.

Our study explored perspectives about food safety across 6 diverse LMICs to expand our understanding of how consumers’ food-safety concerns influence subsequent food-choice behaviors and offer insight into how individual perspectives (i.e., constructed meanings, knowledge, and concerns about food safety) may ultimately affect behaviors around food choice. Thoroughly evaluating food-safety perspectives requires attending to how food choices are influenced by people’s perspectives about the food in their environment. The findings from this study highlight how food-safety concerns emerge from the perspectives consumers construct of their interactions with food throughout the food value chain in their lived food environments. The connections consumers developed to engender assurance in the safety of their food show how the interactions between the individual- and societal-level elements that coalesce to form consumers’ perspectives of food safety are important for shaping consumers’ food-choice behaviors. Failing to consider consumers’ perspectives about food safety, particularly, how their food-safety concerns influence their food-choice behaviors, could exacerbate poor health and development outcomes seen in many LMICs. Further research examining how consumers’ perspectives engender assurance about the safety of their foods is needed to effectively develop programs and policies aiming to improve the safety of food.

## Funding

Funded by the Drivers of Food Choice Competitive Grants Program, which was funded by the United Kingdom Government’s Foreign, Commonwealth & Development Office, and the Bill & Melinda Gates Foundation INV-008124, and managed by the University of South Carolina, Arnold School of Public Health, USA.

## Author disclosures

SI, SVC, EAF, SB, SS, and EK were associated with the subgranting institution that administered funding to the Drivers of Food Choice grantees. SW-H, SN, MH, PD-S, RA, AL, CLP, BK, SAB, and MN led the projects.

## Disclaimer

The views expressed do not necessarily reflect the United Kingdom Government’s official policies. The funders did not influence the design, implementation, analysis, or interpretation of the data presented in this manuscript.

## Author Contribution

The authors’ responsibilities were as follows – SVC, CEB: conceptualized the design; SW-H, SN, MH, PD-S, RA, AL, CLP, BK, SAB, MN: contributed to the conceptualization of the design; SW-H, SN, MH, PD-S, RA, AL, CLP, BK, SAB, MN: contributed to data collection; SI, SVC, CEB: designed the research; SI, SVC, SB, SS, EK: analyzed the data; EAF, CEB: provided feedback on the analysis; SI: led the writing of the manuscript; SI, EAF, CEB: had primary responsibility for the final content; and all authors: reviewed and contributed to the manuscript drafts and read and approved the final manuscript.

## Data Availability

The data described in the manuscript, code book, and analytic code will be made publicly and freely available without restriction at https://dataverse.harvard.edu/dataverse/DFC.
